# Biomechanical Comparison of Pedicle Screw Augmented with Different Volumes of Polymethylmethacrylate in Osteoporotic and Severely Osteoporotic Synthetic Bone Blocks in Primary Implantation: An Experimental Study

**DOI:** 10.1155/2016/9627504

**Published:** 2016-01-17

**Authors:** Da Liu, Xiao-jun Zhang, Dong-fa Liao, Jiang-jun Zhou, Zhi-qiang Li, Bo Zhang, Cai-ru Wang, Wei Lei, Xia Kang, Wei Zheng

**Affiliations:** ^1^Department of Orthopaedics, Chengdu Military General Hospital, No. 270, Rongdu Avenue, Jinniu District, Chengdu, Sichuan 610083, China; ^2^Department of Orthopaedics, People's Hospital of Tongchuan, No. 12, Jiankang Road, Tongchuan, Shaanxi 727000, China; ^3^Department of Orthopaedics, 184 Hospital of Nanjing Military Region, No. 4, Hudong Street, Yingtan, Jiangxi 335000, China; ^4^Department of Orthopaedics, Xijing Hospital, Fourth Military Medical University, No. 15, Changle West Road, Xi'an, Shaanxi 710032, China

## Abstract

This study was designed to compare screw stabilities augmented with different volumes of PMMA and analyze relationship between screw stability and volume of PMMA and optimum volume of PMMA in different bone condition. Osteoporotic and severely osteoporotic synthetic bone blocks were divided into groups A0-A5 and B0-B5, respectively. Different volumes of PMMA were injected in groups A0 to A5 and B0 to B5. Axial pullout tests were performed and *F*_max_ was measured. *F*_max_ in groups A1-A5 were all significantly higher than group A0. Except between groups A1 and A2, A3 and A4, and A4 and A5, there were significant differences on *F*_max_ between any other two groups. *F*_max_ in groups B1-B5 were all significantly higher than group B0. Except between groups B1 and B2, B2 and B3, and B4 and B5, there were significant differences on *F*_max_ between any other two groups. There was significantly positive correlation between *F*_max_ and volume of PMMA in osteoporotic and severely osteoporotic blocks. PMMA can significantly enhance pedicle screw stability in osteoporosis and severe osteoporosis. There were positive correlations between screw stability and volume of PMMA. In this study, injection of 3 mL and 4 mL PMMA was preferred in osteoporotic and severely osteoporotic blocks, respectively.

## 1. Introduction

Transpedicular screw fixation has been widely used in treating degenerative disorders, unstable fractures, and deformities and tumors of the spine [1–4]. However, osteoporosis severely influences the binding strength of the interface between screws and bone and decreases the holding strength of the screws, which usually results in screw loosening, migration, or back-out [5]. Severe osteoporosis increases the need for pedicle screw fixation strength and thus has long been one of the contraindications for spinal internal fixation.

To effectively improve pedicle screw stability in the setting of compromised bone, many researchers have used polymethylmethacrylate (PMMA) to enhance fixation strength [6–18]. There are marked differences for various volumes of injected PMMA and the screw stability that each provides. However, there were little biomechanical comparisons of pedicle screw stabilities augmented with different volumes of injected PMMA in osteoporosis and severe osteoporosis. Moreover, there were little studies on the relationship between screw stability and volume of injected PMMA in osteoporosis and severe osteoporosis [6–14, 16, 17].

This study was designed to compare the pedicle screw stabilities augmented with different volumes of PMMA in different bone conditions which were simulated using synthetic bone blocks with different density. It was also performed to analyze the relationship between screw stability and volume of PMMA and study the preferred volume of injected PMMA in different degree of osteoporosis.

## 2. Materials and Methods

### 2.1. Experiment Materials

Synthetic bone blocks (model 1522-507# and 1522-505#, Pacific Research Laboratory Inc., Vashon Island, WA, USA) were used as a substitute for cadaveric spinal bone in this study because of its consistent and homogeneous structural properties [19, 20]. The synthetic bone was supplied in rectangular shape (test block) with the dimension of 13 cm × 18 cm × 4 cm, and the material was open-cell rigid polyurethane foam with density 0.12 g/cm^3^ and 0.09 g/cm^3^, respectively, which was used to simulate human vertebral cancellous bone with osteoporosis and severe osteoporosis [13, 16–18]. The cell structure is over 95% open, and the cell size is 1.5~2.5 mm [13, 16–20]. Their compressive strength and compressive modulus are 0.28 MPa and 18.6 MPa for 1522-507# blocks and 0.11 MPa and 6.2 MPa for 1522-505# blocks, respectively. The pedicle screws are identical with a length of 45.0 mm, an outer diameter of 6.5 mm, and a core diameter of 4.5 mm, which were made of titanium alloy (Medtronic-Weigao Orthopedic Device, Shandong, China). PMMA (CEMEX, TECRES, Verona, Italy) was used for screw augmentation including cement powder and cement solution.

### 2.2. Experimental Procedures

To carry out the test, firstly, test samples of 13 cm × 9 cm × 4 cm were extracted from the whole blocks. Forty-eight 1522-507# synthetic bone blocks were randomly divided into group A0 to group A5 (*n* = 8). Forty-eight 1522-505# synthetic bone blocks were randomly divided into group B0 to group B5 (*n* = 8). Each hole was tapped using a 4.5 mm tap into depth with 45 mm (Medtronic-Weigao Orthopedic Device, Shandong, China). Different volumes of PMMA (0 mL, 1 mL, 2 mL, 3 mL, 4 mL, and 5 mL) were retrograde injected into the pilot hole in groups A0 and B0, groups A1 and B1, groups A2 and B2, groups A3 and B3, groups A4 and B4, and groups A5 and B5, respectively. The bone cement powder was mixed with the cement solution at a ratio of 2 : 1 according to manufacturer's recommendations. Then we connected the syringe and injection sheath and injected the PMMA until it appeared at the tip of the sheath. We used visual observation and palpation to ensure that the PMMA had pushed out the air in the sheath cavity and to evaluate the viscosity of the PMMA. When the PMMA reached a toothpaste-like consistency, we inserted the sheath into the hole. PMMA was injected through the sheath into the hole and slowly diffused towards surrounding synthetic bone material under the continue pressure. Screw was inserted into the hole immediately after injection of PMMA. All screws were inserted by hand using a ratcheted screwdriver until the hub of the screw was firmly seated against the surface of test blocks. After complete solidification of PMMA, all vertebrae were taken for X-ray examination and the distribution of PMMA around screw was observed.

### 2.3. Axial Pullout Tests

After X-ray examination, the axial pullout test was performed with MTS 858 Material Testing System (MTS System, Minneapolis, MN, USA). A tensile load was gradually applied to the head of the screw at a constant speed of 5 mm/min until the screw was pulled out from the test block. The load and displacement data collected in real time at 50 Hz were used to obtain the load-displacement curve. On the curve, the maximum pullout strength (*F*_max⁡_) was defined as the highest point which was also the inflection point between buildup curve and descending curve [21–24]. After axial pullout tests, the destructions of blocks and remains of screws were observed.

### 2.4. Statistical Analysis

Statistical analysis was performed with SPSS for Windows (version 16.0; SPSS Inc., Chicago, IL, USA). The data were expressed as mean ± standard deviation. The two-way ANOVA and LSD test were used to detect the differences in *F*_max⁡_ among twelve groups and between any two groups. Relationships between the *F*_max⁡_ and the volume of injected PMMA were assessed using linear regression analyses. Statistical significance was defined as *P* < 0.05.

## 3. Results

### 3.1. X-Ray Examination

**Figure 1:**
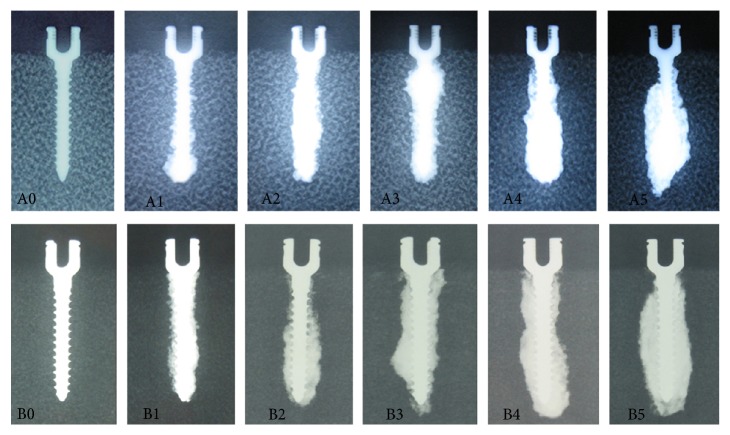
X-ray examination of six groups in 1522-507# blocks and 522-505# blocks. “A0,” “A1,” “A2,” “A3,” “A4,” and “A5” indicate group A0, group A1, group A2, group A3, group A4, and group A5, respectively. “B0,” “B1,” “B2,” “B3,” “B4,” and “B5” indicate group B0, group B1, group B2, group B3, group B4, and group B5, respectively.

The distribution of PMMA and screw was clearly shown in X-ray examination (Figure 1). The density of 1522-507# blocks in X-ray was obviously higher than that of 1522-505# blocks. There were similar distribution patterns of PMMA around screw between that in 1522-507# blocks and 1522-505# blocks. In groups A0 and B0, the screw was surrounded directly by synthetic bone and no PMMA was found around screw. In groups A1–A5 and groups B1–B5, the screw was wrapped up by PMMA totally. The PMMA was nearly evenly distributed around screw, which obviously improved the local density around track. From group A1 to group A5 and from group B1 to group B5, it was found with the gradually broadening distribution range of PMMA around screw.

### 3.2. Axial Pullout Tests

**Figure 2:**
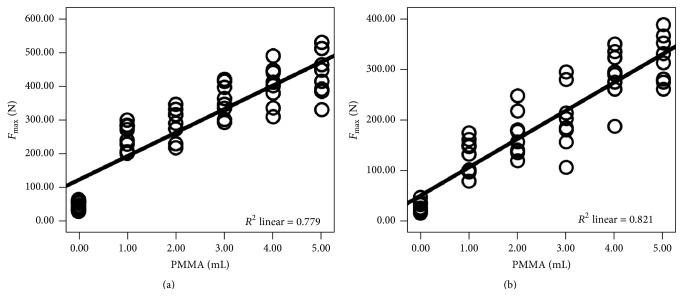
The correlation between *F*_max⁡_ and volume of injected PMMA in 1522-507# and 1522-505# blocks. (a) and (b) indicate 1522-507# and 1522-505# blocks, respectively.

*F*_max⁡_ in group A0 to group A5 were 48.75 ± 12.54 N, 252.12 ± 37.59 N, 290.50 ± 46.91 N, 359.25 ± 48.95 N, 402.38 ± 59.50 N, and 434.62 ± 67.11 N, respectively. *F*_max⁡_ in groups A1–A5 were all significantly higher than that in group A0 (*P* < 0.05). There were 417.2%, 495.9%, 636.9%, 725.4%, and 791.5% increments on *F*_max⁡_ in groups A1–A5 compared with that in group A0, respectively. Except the no significant differences on *F*_max⁡_ between groups A1 and A2, between groups A3 and A4, and between groups A4 and A5 (*P* = 0.102, *P* = 0.067, and *P* = 0.169), there were significant differences on *F*_max⁡_ between any other two groups (*P* < 0.05). As shown in Figure 2(a), there was strong positive statistically significant correlation between *F*_max⁡_ and the volume of injected PMMA (*r* = 0.882, *R* ^2^ = 0.779, and *P* < 0.05).

*F*_max⁡_ in group B0 to group B5 were 30.63 ± 10.45 N, 131.12 ± 34.06 N, 172.38 ± 43.41 N, 202.75 ± 61.71 N, 289.62 ± 51.00 N, and 320.88 ± 46.22 N, respectively. *F*_max⁡_ in groups B1–B5 were all significantly higher than that in group B0 (*P* < 0.05). There were 328.08%, 462.78%, 561.93%, 845.54%, and 947.60% increments on *F*_max⁡_ in groups B1–B5 compared with that in group B0, respectively. Except the no significant differences on *F*_max⁡_ between groups B1 and B2, between groups B2 and B3, and between groups B4 and B5 (*P* = 0.079, *P* = 0.195, and *P* = 0.182), there were significant differences on *F*_max⁡_ between any other two groups (*P* < 0.05). As shown in Figure 2(b), there was strong positive statistically significant correlation between *F*_max⁡_ and the volume of injected PMMA (*r* = 0.906, *R* ^2^ = 0.821, and *P* < 0.05).

**Figure 3:**
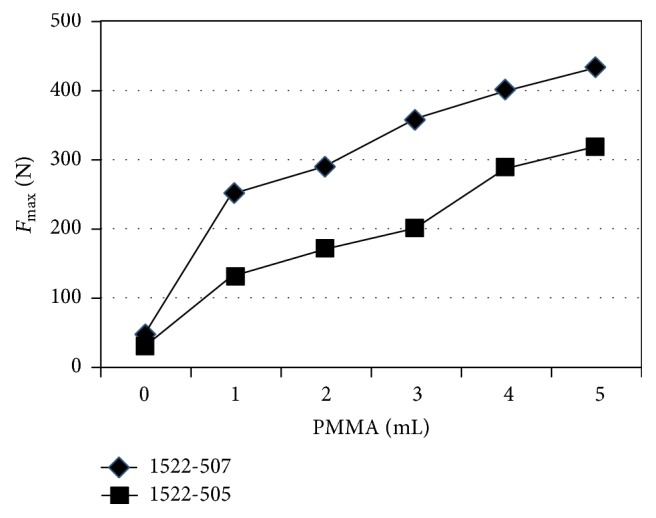
Comparison of *F*_max⁡_ of pedicle screw augmented with different volume of PMMA in different blocks.

As shown in Figure 3, in augmentation with the same volume of PMMA, the *F*_max⁡_ in groups B0–B5 in 1522-505# blocks was significantly decreased (37.17%, 47.99%, 40.66%, 43.56%, 28.02%, and 26.17%, resp.) compared with the *F*_max⁡_ in groups A0–A5 in 1522-507# blocks. Except no significant difference on *F*_max⁡_ between groups A0 and B0 (*P* = 0.438), there were significant difference on *F*_max⁡_ between any two groups with same volume of injected PMMA (*P* < 0.05). The *F*_max⁡_ in group A0 was significantly lower than those in groups B1, B2, B3, B4, and B5 (*P* = 0.001, 0.000, 0.000, 0.000, and 0.000, resp.) and not significantly different from that in groups B0 (*P* = 0.438). The *F*_max⁡_ in group A1 was significantly higher than that in groups B0, B1, B2, and B3 (*P* = 0.000, 0.000, 0.001, and 0.037, resp.), significantly lower than that in group B5 (*P* = 0.004), and without significant difference compared with that in group B4 (*P* = 0.110). The *F*_max⁡_ in group A2 was significantly higher than that in groups B0, B1, B2, and B3 (*P* = 0.000, 0.000, 0.000, and 0.000, resp.) and without significant difference compared with that in both group B4 and group B5 (*P* = 0.970 and 0.195, resp.). The *F*_max⁡_ in group A3 was significantly higher than that in groups B0, B1, B2, B3, and B4 (*P* = 0.000, 0.000, 0.000, 0.000, and 0.004, resp.) and without significant difference compared with that in group B5 (*P* = 0.102). The *F*_max⁡_ in both groups A4 and A5 were significantly higher than that in groups B0, B1, B2, B3, B4, and B5 (*P* < 0.05).

### 3.3. Observation of Destruction of Blocks and Remains of Screws

**Figure 4:**
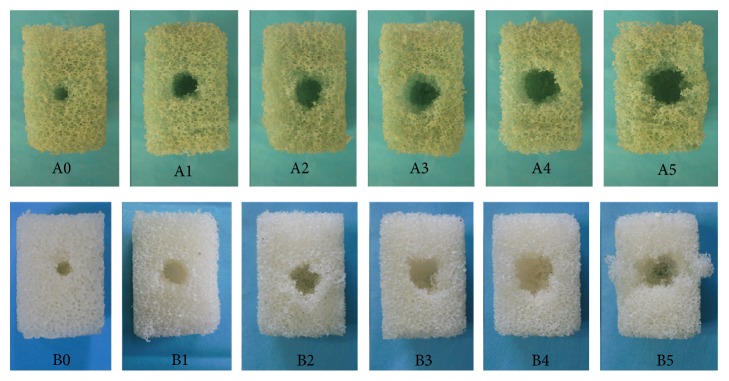
The destruction of 1522-507# blocks and 1522-505# blocks after pullout test. “A0,” “A1,” “A2,” “A3,” “A4,” and “A5” indicate group A0, group A1, group A2, group A3, group A4, and group A5, respectively. “B0,” “B1,” “B2,” “B3,” “B4,” and “B5” indicate group B0, group B1, group B2, group B3, group B4, and group B5, respectively.

As shown in Figure 4, there were different extents of destruction of block in different groups through visual observation. There was the most slight destruction in both group A0 and group B0 and most severe destruction in both group A5 and group B5. The extents of destruction of block were increased from groups A0 to A5 and from B0 to B5. There was similar extent of destruction through visual observation between two groups with the same volume of injected PMMA.

**Figure 5:**
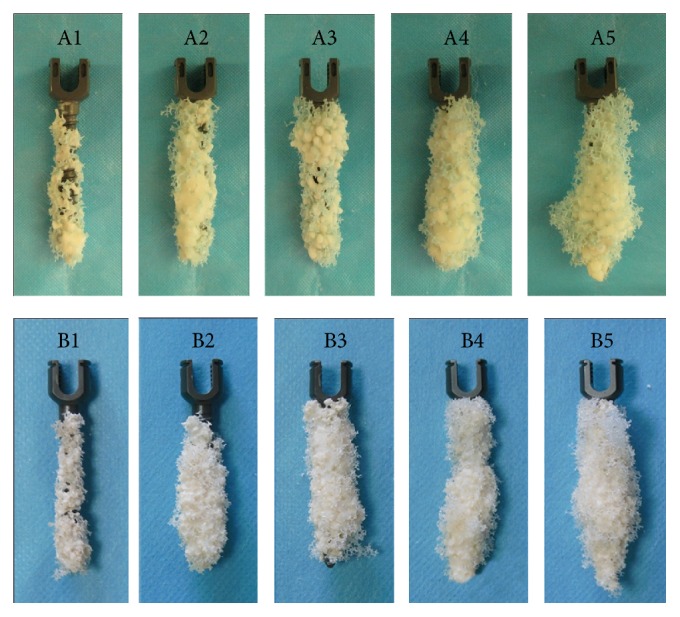
The remains of screw after pullout from 1522-507# blocks and 1522-505# blocks. “A1,” “A2,” “A3,” “A4,” and “A5” indicate group A1, group A2, group A3, group A4, and group A5, respectively. “B1,” “B2,” “B3,” “B4,” and “B5” indicate group B1, group B2, group B3, group B4, and group B5, respectively.

As showed in Figure 5, all PMMA-augmented pedicle screws were tightly wrapped by PMMA and pulled out together with PMMA in groups A1–A5 and groups B1–B5, and all the destructions were found between screw/PMMA composite and polyurethane material. The scope of PMMA surrounding screw was gradually increasing from groups A1 to A5 and from groups B1 to B5. There was similar scope of PMMA surrounding screw between two groups with the same volume of injected PMMA.

## 4. Discussion

Effectively improving pedicle screw stability in osteoporosis has always been a tough problem for spine surgeons. With the excellent mechanical strength, PMMA was used to augment screw fixation in more and more studies. Earlier, many researchers directly injected PMMA into the pilot to improve screw stability. With the development of study, more and more researchers injected PMMA through different-designed injectable screw to enhance screw fixation. However, there was significant variation in the volumes of PMMA injected and in screw stability across studies. As shown in Table 1, the volume of PMMA injected ranged from 0.8 to 8.0 mL and incremental screw stability varied markedly from 28.7% to 1031%.

Frankel et al. [7] injected PMMA (mean volume, 3.7 mL; range, 2–8.0 mL) through a novel fenestrated bone tap into the vertebral body between T5 and L5 to enhance pedicle screw stability in osteoporotic human cadaveric specimens. After PMMA augmentation, the *F*_max⁡_ of the pedicle screw increased in primary and salvage procedures by 119% and 162%, respectively, but pullout strength did not significantly change with increased cement usage between the low-cement group (≤2.8 mL/pedicle) and the high-cement group (≥5.5 mL/pedicle). Paré et al. [15] injected different volumes of PMMA into vertebrae through fenestrated pedicle screws (0.5, 1.0, and 1.5 mL in thoracic vertebrae, and 1.5, 2.0, and 2.5 mL in lumbar vertebrae). Except for the screw receiving 0.5 mL of PMMA in thoracic vertebrae, the *F*_max⁡_ of the augmented fenestrated screws was significantly increased compared with non-PMMA-augmented pedicle screws for the screws receiving 1.0 mL (186%) and 1.5 mL of PMMA (158%) in the thoracic vertebrae. Statistically significant increases were observed for the screws receiving 1.5 mL (264%), 2.0 mL (221%), and 2.5 mL (198%) of injected cement in the lumbar vertebrae. There was no significant difference, however, at higher volumes of cement. They found that screw stability did not increase with incremental increases in PMMA volume. However, they did not analyze the relationship between screw stability and volume of PMMA in their study. Chen et al. [18] found that injection of 1, 2, or 3 mL of PMMA significantly improved the screw stability by 259%, 508%, and 715%, respectively, in severely osteoporotic blocks compared with non-PMMA-augmented pedicle screws, and there were significant differences between any 2 groups. With every 1 mL increment of PMMA, there was a significant improvement in fixation strength.

All of those studies show that there are significant differences in the screw stability provided by various volumes of PMMA. Nevertheless, most comparative studies of different volumes of PMMA mainly focused on injection through fenestrated screws; few studies have focused on the traditional injection method. In studies that did focus on the traditional method, there was no comparison of screw stability for varying volumes of PMMA and no correlative analysis of screw fixation strength and PMMA volume, especially in different degrees of osteoporosis. Thus, our study was designed to fill that research void. As we knew, it was very difficult to obtain osteoporotic and severe osteoporotic cadaveric spinal samples. In this study, we choose synthetic bone (model 1522-507# and 1522-505#) made from polyurethane foam as experiment samples. It has homogeneous structural properties and over 95% open cell structure, which is suitable for modeling osteoporotic and severe osteoporotic cancellous bone and injection of PMMA and has been used in some biomechanical study [13, 16–18].

Our previous study proved that injection sheath worked well and was easy to use. But we have applied for a patent on this sheath in China, so we cannot publicly share images of the sheath yet in the published papers. In this study, under the continuous pressure from injection PMMA was slowly diffusing toward the surrounding pores along the total length of hole. PMMA was proved nearly evenly distributing around screw in both previous studies [17, 25–27] and this study. In order to diminish the influence on screw stability caused by different distribution of PMMA, we used the same injection method and hoped to keep the similar and even distribution of PMMA around screw as possible as we could. In this study, we performed comparison of screw stabilities augmented with different volumes of PMMA in osteoporotic and severe osteoporotic synthetic bone blocks. From the related references, we found 3 mL was the most frequently used volume of injected PMMA. Considering 3 mL as median, we designed several groups with different volumes of PMMA and 1 mL increment between any consecutive two groups in this study and planned a preliminary comparison and found the preferred volume of injected PMMA.

PMMA (showing high density in Figure 1) was found wrapping up screw with nearly uniform distribution in osteoporotic and severe osteoporotic blocks, improving the local density around screw, adhering screw to synthetic bone tightly, and enhancing holding strength between screw and synthetic bone. It was proved in both X-ray examination and remains of screws that the scope of PMMA around screw was broadening with the volume of injected PMMA increasing from groups A1 to A5 and from groups B1 to B5. Biomechanical tests proved that augmentation with different volumes of PMMA could all significantly increase fixation strength of pedicle screw compared with no-augmented screw in both osteoporosis and severe osteoporosis. All screws were tightly surrounded with PMMA and pulled out together with PMMA in groups A1–A5 and groups B1–B5, and all the types of destruction were found between screw/PMMA composite and polyurethane material. With the increasing volumes of injected PMMA, moreover, the overall diameter of the screw/PMMA composite and the contact area between screw/PMMA composite and surrounding synthetic bone all increased gradually (Figure 1), which may lead to gradually enhancing the shearing force between screw/PMMA and synthetic bone during the pullout test in theory. In biomechanical tests, the screw stabilities increased synchronously with the volume of injected PMMA increasing from 0 mL to 5 mL. There was strong positive statistically significant correlation between screw stability and volume of PMMA in both osteoporosis and severe osteoporosis. The extents of destruction in block obviously increased from group A0 to group A5 and from group B0 to group B5 (Figure 5), which reflected that it needs more and more energy to pull out screw and showed more and more stability from group A0 to group A5 and from group B0 to group B5.

Though there was no significant difference on *F*_max⁡_ of nonaugmented screws in osteoporotic block compared with that in severe osteoporotic block, the *F*_max⁡_ of PMMA-augmented screws in osteoporotic block was markedly higher than that in severe osteoporotic block augmented with the same volume of PMMA. It was demonstrated through two-way ANOVA and LSD test that the holding force is directly correlated to both the density of block and volume of injected PMMA. In comparative study among different volume of PMMA in the same density block, it was revealed that screw stability positively correlated with volume of injected PMMA in both density blocks. Though there were 15.2%, 12.0%, and 8.0% increments on screw stabilities in osteoporotic block with increasing volume of injected PMMA from 1 mL to 2 mL, from 3 mL to 4 mL, and from 4 mL to 5 mL, all these increments were not with statistical significance (*P* = 0.102, *P* = 0.067, and *P* = 0.169). As the same, though there were 31.5%, 17.6%, and 10.8% increments on screw stabilities in severe osteoporotic block with increasing volume of injected PMMA from 1 mL to 2 mL, from 2 mL to 3 mL, and from 4 mL to 5 mL, all those increments were not with statistical significance (*P* = 0.079, *P* = 0.195, and *P* = 0.182). Chen et al. [18] revealed that injection of 1 mL, 2 mL, and 3 mL PMMA could all significantly improve the screw stability in sever osteoporotic block and there were significant differences between any two groups, which was different from the result in our study. With every increment of 1 mL PMMA, there was all significant improvement on fixation strength. It may be related to the different distribution of PMMA around screw, with most PMMA around the screw tip in Chen et al.'s study [18] and most PMMA around the whole screw in our study. In the present study, it is demonstrated that although screw stability positively correlated with volume of injected PMMA, it did not mean that increasing volume of injected PMMA certainly significantly improved screw stability. Increasing volume of PMMA within a certain range did not significantly improve screw fixation strength but would increase the leakage risk of PMMA. In the present study, we preferred the injection of 3 mL PMMA in osteoporotic block and injection of 4 mL PMMA in severe osteoporotic block, respectively.

The 1522-507# and 1522-505# synthetic bone blocks were used to simulate human vertebral cancellous bone with osteoporosis and severely osteoporosis, respectively, in this study. The synthetic bone block has consistent and homogeneous structural properties. So that the synthetic bone block was just used to simulate but could not replace the vertebral samples. In order to simulate the real condition in operation in clinic, therefore, it is necessary to perform the comparative study in osteoporotic and severe osteoporotic lumbar vertebrae in future study. In this study, we not only found the relationship between screw stability and volume of PMMA and the preferred volume of injected PMMA in synthetic bone blocks with different density, but also provided the feasibility for the future study in vertebral samples. The result in this study also provided the guideline for the injected volume of PMMA in vertebral body in the future. As we knew, there were different microstructures of bone tissue in different vertebral bodies. There were also different microstructures of cancellous bone in different regions in one vertebral body. For this reason, we cannot ensure the exact same distribution and interdigitation of PMMA to the surrounding bone tissue by vertebra to vertebra. That may bring influence on the fixation strength of pedicle screw. In order to study relationship between the different distribution of PMMA and fixation strength, we also had designed several injection sheaths with different lateral holes. With the same volume of injected PMMA, we found varying distribution of PMMA and varying screw stability. The results of that particular experiment are still being interpreted and have not been submitted to any journal.

## 5. Conclusions

In this study, PMMA can significantly enhance stability of pedicle screw in both osteoporotic block and severely osteoporotic synthetic bone block. There were strong positive statistically significant correlations between screw stability and the volume of PMMA, and the screw stability was increased with the increment in the volume of PMMA. Within a certain range, nevertheless, increasing the volume of PMMA does not significantly improve screw stability. In this study, injection of 3 mL PMMA and injection of 4 mL PMMA were considered as preferred choice in osteoporosis and severe osteoporosis, respectively.

## Figures and Tables

**Table 1:** The volume of injected PMMA reported in references.

References	Type of study	The volume of injected PMMA	The increment of pedicle screw stability
Fransen [6]	Clinical study in patients with osteoporosis and severe osteoporosis (T12-L1, L3–L5)	1.5 mL	

Frankel et al. [7]	Study in vitro in osteoporotic thoracic and lumbar vertebrae (T5-L5)	2.0~8.0 mL	The first surgery 119% The revision surgery 162%

Becker et al. [8]	Study in vitro in osteoporotic lumbar vertebrae (L1–L4)	2.0 mL	79%

Chang et al. [9]	Clinical study in patients with osteopenia, osteoporosis, and severe osteoporosis (T2-S1)	3.0 mL (lumbar vertebrae) and 2–2.5 mL (thoracic vertebrae and S1)	

Moon et al. [10]	Clinical study in patients with osteoporosis and severe osteoporosis (T2-S1)	1.7~2.0 mL	

Waits et al. [11]	Study in vitro in osteopenia lumbar vertebrae	2.5 mL	

Blattert et al. [12]	Study in vitro in osteoporotic thoracic and lumbar vertebrae (T9-L4)	1.5 mL	

Chen et al. [13]	Study in vitro in severe osteoporotic synthetic bone block	3 mL	

Bullmann et al. [14]	Study in vitro in osteoporotic thoracic and lumbar vertebrae (T7-L3)	0.8–2.0 mL	28.7%

Paré et al. [15]	Study in vitro in osteoporotic thoracic and lumbar vertebrae (T7-L5)	Thoracic vertebrae (0.5 mL, 1.0 mL, and 1.5 mL) and lumbar vertebrae (1.5 mL, 2.0 mL, and 2.5 mL)	Thoracic vertebrae, 186% (1.0 mL) and 158% (1.5 mL), and lumbar vertebrae, 264% (1.5 mL), 221% (2.0 mL), amd 198% (2.5 mL)

Chen et al. [16]	Study in vitro in severe osteoporotic synthetic bone block	3 mL	1031%, 817.1%, 902.4%, and 609.5%

Liu et al. [17]	Study in vitro in osteoporotic synthetic bone block	2.5 mL	448%

Chen et al. [18]	Study in vitro in severe osteoporotic synthetic bone block	1 mL, 2 mL, and 3 mL	259% (1.0 mL), 508% (2.0 mL), and 715% (3.0 mL)

## References

[1] Kaymaz B., Demirkiran G., Ayvaz M., Akel I., Acaroğlu E., Alanay A. (2014). Treatment of thoracolumbar burst fractures using combined pedicle screw-laminar hook fixation. *Acta Orthopaedica et Traumatologica Turcica*.

[2] Wang L., Li J., Wang H. (2014). Posterior short segment pedicle screw fixation and TLIF for the treatment of unstable thoracolumbar/lumbar fracture. *BMC Musculoskeletal Disorders*.

[3] Heo D. H., Cho Y. J., Cho S. M., Choi H. C., Kang S. H. (2012). Adjacent segment degeneration after lumbar dynamic stabilization using pedicle screws and a nitinol spring rod system with 2-year minimum follow-up. *Journal of Spinal Disorders and Techniques*.

[4] Jindal N., Sankhala S. S., Bachhal V. (2012). The role of fusion in the management of burst fractures of the thoracolumbar spine treated by short segment pedicle screw fixation: a prospective randomised trial. *The Journal of Bone & Joint Surgery—British Volume*.

[5] Reitman C. A., Nguyen L., Fogel G. R. (2004). Biomechanical evaluation of relationship of screw pullout strength, insertional torque, and bone mineral density in the cervical spine. *Journal of Spinal Disorders and Techniques*.

[6] Fransen P. (2007). Increasing pedicle screw anchoring in the osteoporotic spine by cement injection through the implant. Technical note and report of three cases. *Journal of Neurosurgery Spine*.

[7] Frankel B. M., D'Agostino S., Wang C. (2007). A biomechanical cadaveric analysis of polymethylmethacrylate-augmented pedicle screw fixation. *Journal of Neurosurgery: Spine*.

[8] Becker S., Chavanne A., Spitaler R. (2008). Assessment of different screw augmentation techniques and screw designs in osteoporotic spines. *European Spine Journal*.

[9] Chang M.-C., Liu C.-L., Chen T.-H. (2008). Polymethylmethacrylate augmentation of pedicle screw for osteoporotic spinal surgery: a novel technique. *Spine*.

[10] Moon B. J., Cho B. Y., Choi E. Y., Zhang H. Y. (2009). Polymethylmethacrylate-augmented screw fixation for stabilization of the osteoporotic spine: a three-year follow-up of 37 patients. *Journal of Korean Neurosurgical Society*.

[11] Waits C., Burton D., McIff T. (2009). Cement augmentation of pedicle screw fixation using novel cannulated cement insertion device. *Spine*.

[12] Blattert T. R., Glasmacher S., Riesner H.-J., Josten C. (2009). Revision characteristics of cement-augmented, cannulated-fenestrated pedicle screws in the osteoporotic vertebral body: a biomechanical in vitro investigation. Technical note. *Journal of Neurosurgery Spine*.

[13] Chen L.-H., Tai C.-L., Lai P.-L. (2009). Pullout strength for cannulated pedicle screws with bone cement augmentation in severely osteoporotic bone: influences of radial hole and pilot hole tapping. *Clinical Biomechanics*.

[14] Bullmann V., Schmoelz W., Richter M., Grathwohl C., Schulte T. L. (2010). Revision of cannulated and perforated cement-augmented pedicle screws: a biomechanical study in human cadavers. *Spine*.

[15] Paré P. E., Chappuis J. L., Rampersaud R. (2011). Biomechanical evaluation of a novel fenestrated pedicle screw augmented with bone cement in osteoporotic spines. *Spine*.

[16] Chen L.-H., Tai C.-L., Lee D.-M. (2011). Pullout strength of pedicle screws with cement augmentation in severe osteoporosis: a comparative study between cannulated screws with cement injection and solid screws with cement pre-filling. *BMC Musculoskeletal Disorders*.

[17] Liu D., Shi L., Lei W. (2013). Biomechanical comparison of expansive pedicle screw and polymethylmethacrylate-augmented pedicle screw in osteoporotic synthetic bone in primary implantation: an experimental study. *Journal of Spinal Disorders and Techniques*.

[18] Chen Y.-L., Chen W.-C., Chou C.-W. (2014). Biomechanical study of expandable pedicle screw fixation in severe osteoporotic bone comparing with conventional and cement-augmented pedicle screws. *Medical Engineering and Physics*.

[19] Kim Y.-Y., Choi W.-S., Rhyu K.-W. (2012). Assessment of pedicle screw pullout strength based on various screw designs and bone densities—an ex vivo biomechanical study. *Spine Journal*.

[20] Ying S.-H., Kao H.-C., Chang M.-C., Yu W.-K., Wang S.-T., Liu C.-L. (2012). Fixation strength of PMMA-augmented pedicle screws after depth adjustment in a synthetic bone model of osteoporosis. *Orthopedics*.

[21] Inceoglu S., Ferrara L., McLain R. F. (2004). Pedicle screw fixation strength: pullout versus insertional torque. *The Spine Journal*.

[22] Yüksel K. Z., Adams M. S., Chamberlain R. H. (2007). Pullout resistance of thoracic extrapedicular screws used as a salvage procedure. *The Spine Journal*.

[23] Rohmiller M. T., Schwalm D., Glattes R. C., Elalayli T. G., Spengler D. M. (2002). Evaluation of calcium sulfate paste for augmentation of lumbar pedicle screw pullout strength. *The Spine Journal*.

[24] Cook S. D., Salkeld S. L., Stanley T., Faciane A., Miller S. D. (2004). Biomechanical study of pedicle screw fixation in severely osteoporotic bone. *The Spine Journal*.

[25] Liu D., Zhang Y., Lei W. (2014). Comparison of 2 kinds of pedicle screws in primary spinal instrumentation: biomechanical and interfacial evaluations in sheep vertebrae in vitro. *Journal of Spinal Disorders and Techniques*.

[26] Liu D., Zhang Y., Zhang B. (2013). Comparison of expansive pedicle screw and polymethylmethacrylate-augmented pedicle screw in osteoporotic sheep lumbar vertebrae: biomechanical and interfacial evaluations. *PLoS ONE*.

[27] Liu D., Wu Z.-X., Pan X.-M. (2011). Biomechanical comparison of different techniques in primary spinal surgery in osteoporotic cadaveric lumbar vertebrae: expansive pedicle screw versus polymethylmethacrylate-augmented pedicle screw. *Archives of Orthopaedic and Trauma Surgery*.

